# Socioeconomic Deprivation: An Important, Largely Unrecognized Risk Factor in Primary Prevention of Cardiovascular Disease

**DOI:** 10.1161/CIRCULATIONAHA.122.060042

**Published:** 2022-06-24

**Authors:** Dorien M. Kimenai, Leah Pirondini, John Gregson, David Prieto, Stuart J. Pocock, Pablo Perel, Tilly Hamilton, Paul Welsh, Archie Campbell, David J. Porteous, Caroline Hayward, Naveed Sattar, Nicholas L. Mills, Anoop S.V. Shah

**Affiliations:** British Heart Foundation Centre for Cardiovascular Science (D. M.K., T.H., N.L.M.), University of Edinburgh, United Kingdom.; Centre for Genomic and Experimental Medicine (A.C., D.J.P.), University of Edinburgh, United Kingdom.; Medical Research Council Human Genetics Unit (C.H.), University of Edinburgh, United Kingdom.; Institute of Genetics and Cancer, Usher Institute (N.L.M.), University of Edinburgh, United Kingdom.; Department of Medical Statistics (L.P., J.G., S.J.P.), London School of Hygiene & Tropical Medicine, United Kingdom.; Department of Non-communicable Disease Epidemiology (D.P., P.P., A.S.V.S.), London School of Hygiene & Tropical Medicine, United Kingdom.; Institute of Cardiovascular & Medical Sciences, University of Glasgow, United Kingdom (P.W., N.S.).

**Keywords:** calibration, cardiovascular disease, risk assessment, social class

## Abstract

**Background:**

Socioeconomic deprivation is associated with higher cardiovascular morbidity and mortality. Whether deprivation status should be incorporated in more cardiovascular risk estimation scores remains unclear. This study evaluates how socioeconomic deprivation status affects the performance of 3 primary prevention cardiovascular risk scores.

**Methods:**

The Generation Scotland Scottish Family Health Study was used to evaluate the performance of 3 cardiovascular risk scores with (ASSIGN [Assessing cardiovascular risk using SIGN (Scottish Intercollegiate Guidelines Network) guidelines to ASSIGN preventive treatment]) and without (SCORE2 [Systematic Coronary Risk Evaluation 2 algorithm], Pooled Cohort Equations) socioeconomic deprivation as a covariate in the risk prediction model. Deprivation was defined by Scottish Index of Multiple Deprivation score. The predicted 10-year risk was evaluated against the observed event rate for the cardiovascular outcome of each risk score. The comparison was made across 3 groups defined by the deprivation index score consisting of group 1 defined as most deprived, group 3 defined as least deprived, and group 2, which consisted of individuals in the middle deprivation categories.

**Results:**

The study population consisted of 15 506 individuals (60.0% female, median age of 51). Across the population, 1808 (12%) individuals were assigned to group 1 (most deprived), 8119 (52%) to group 2, and 4708 (30%) to group 3 (least deprived), and 871 (6%) individuals had a missing deprivation score. Risk scores based on models that did not include deprivation status significantly under predicted risk in the most deprived (6.43% observed versus 4.63% predicted for SCORE2 [*P*=0.001] and 6.69% observed versus 4.66% predicted for Pooled Cohort Equations [*P*<0.001]). Both risk scores also significantly overpredicted the risk in the least deprived group (3.97% observed versus 4.72% predicted for SCORE2 [*P*=0.007] and 4.22% observed versus 4.85% predicted for Pooled Cohort Equations [*P*=0.028]). In contrast, no significant difference was demonstrated in the observed versus predicted risk when using the ASSIGN risk score, which included socioeconomic deprivation status in the risk model.

**Conclusions:**

Socioeconomic status is a largely unrecognized risk factor in primary prevention of cardiovascular disease. Risk scores that exclude socioeconomic deprivation as a covariate under- and overestimate the risk in the most and least deprived individuals, respectively. This study highlights the importance of incorporating socioeconomic deprivation status in risk estimation systems to ultimately reduce inequalities in health care provision for cardiovascular disease.

**Clinical Perspective**

**What is New?**

- We report the impact of socioeconomic deprivation on the performance of 3 primary prevention cardiovascular risk scores that are widely used in practice.
- Socioeconomic deprivation status is an important covariate in cardiovascular risk estimation systems, and risk scores that exclude socioeconomic deprivation under- and overestimate risk in the most and least deprived individuals, respectively.

**What Are the Clinical Implications?**

- Socioeconomic status is a largely unrecognized risk factor in primary prevention of cardiovascular disease.
- Our findings highlight the importance of socioeconomic deprivation status as a covariate that needs to be considered in addition to the traditional risk factors to promote equitable health care, particularly in those most deprived.

Socioeconomic deprivation is closely associated with cardiovascular morbidity and mortality.^1–5^ Although the burden from cardiovascular disease has decreased over time, health care inequity by deprivation status has persisted.^6^ Individuals from poorer backgrounds are less likely to receive evidence-based therapy and more likely to experience higher cardiovascular mortality and morbidity.^7^ Although previous research has extensively studied provision and management of therapy in the primary prevention of cardiovascular disease, the incorporation of deprivation status in risk estimation systems in primary care is less clear.

Cardiovascular risk estimation is the cornerstone for primary prevention of cardiovascular disease. Despite socioeconomic status being closely associated with cardiovascular mortality and morbidity, most cardiovascular risk estimation systems do not incorporate deprivation status in prediction modeling.^8,9^ In the United Kingdom, the ASSIGN (assessing cardiovascular risk using SIGN [Scottish Intercollegiate Guidelines Network] guidelines to ASSIGN preventive treatment) and QRISK3 risk scores incorporate deprivation status as a covariate.^10,11^ However, although international guidelines do make reference to deprivation as important risk modifier, key European- and United States–based cardiovascular risk estimation systems do not incorporate deprivation status in prediction modeling.^8,9,12–18^ Risk estimation systems may vary in performance by deprivation status,^19–21^ highlighting the need for specific research to evaluate whether its inclusion in cardiovascular risk estimation systems is warranted.

We compare the predictive ability of risk scores that include (ASSIGN)^10^ or exclude (SCORE2 [Systematic Coronary Risk Evaluation 2 algorithm],^16^ Pooled Cohort Equations [PCE]^13^) socioeconomic deprivation status in prediction modeling using a large contemporary cohort with >10 years of follow-up.

## Methods

Because of the sensitive nature of the data collected for this study, requests to access the dataset from qualified researchers trained in human subject confidentiality protocols should be sent to the Generation Scotland management team at access@generationscotland.org.

### Study Population

We used data from the GS:SFHS (Generation Scotland Scottish Family Health Study). GS:SFHS is a well-phenotyped family-based contemporary cohort that enrolled 24 090 participants aged between 18 and 98 years as previously described.^22,23^ Briefly, individuals between 35 and 65 years old were identified at random from participating general medical practices in Scotland between February 2006 and March 2011. Participants were then asked to identify ≥1 first-degree relatives aged ≥18 years who would also be able to participate.

For this study, participants <30 years of age, or who had cardiovascular disease at baseline, or who did not attend the clinical survey were excluded. Participants completed a health questionnaire, and clinical characteristics were measured using a standardized protocol. Data were collected on age, sex, diabetes, systolic blood pressure, body mass index, family history of cardiovascular disease, smoking status, and rheumatoid arthritis. Total cholesterol, high-density lipoprotein cholesterol, and serum creatinine were measured at the time of collection. Ethical approval for the GS:SFHS study was obtained from the National Health Service Tayside Research Ethics Committee (Research Ethics Committee reference number 05/S1401/89). Study participants provided written informed consent, including linkage to their medical records. The study was conducted according to principles of the Declaration of Helsinki.

### Outcomes

We used the Information Services Division National Health Service record linkage for Scotland to collect nonfatal cardiovascular events and cause-specific death data for each individual from the date of inclusion in the study until the end of August 2021. Information on cause of death was obtained using the National Health Service Central Register. Nonfatal cardiovascular events and cause-specific deaths were classified using the *International Classification of Diseases, Tenth Revision (ICD-10*).

### Socioeconomic Deprivation Status

Socioeconomic deprivation status was determined using the Scottish Index of Multiple Deprivation (SIMD) 2009 score, which is derived from participants’ postal codes and compiled using 7 domains of deprivation (income, employment, education, health, access to services, crime, and housing).^24^ The SIMD score is recalculated every few years, and the scores used are from 2009, at the midpoint of GS:SFHS recruitment. SIMD scores range from 0.94 (least deprived) to 89.89 (most deprived), with quintiles based on the full derivation cohort, reflecting the wider Scottish population. The cutoffs are as follows: SIMD<7.94 (quintile 5), 7.94 ≤SIMD<13.67 (quintile 4), 13.67≤SIMD<20.98 (quintile 3), 20.98≤SIMD<33.81 (quintile 2), and SIMD≥33.81 (quintile 1).^24,25^ For this study, we classified patients into 3 groups using these quintile-based cutoffs: group 1 (most deprived on the basis of quintile 1), group 2 (on the basis of quintiles 2–4), and group 3 (least deprived on the basis of quintile 5).

### Cardiovascular Risk Scores

The performance of ASSIGN, SCORE2, and the PCE risk scores were evaluated.^10,13,16^ Of these, only the ASSIGN risk score includes socioeconomic deprivation as a covariate, and the ASSIGN risk score uses the SIMD score divided by 10 as a covariate in the risk equation. β-Coefficients, centering values, and 10-year baseline survival for each risk score were extracted. Outcomes were based on *ICD-10* diagnostic classification, and outcomes in the GS:SFHS cohort were mapped to the outcomes of each risk score using *ICD-10* codes. For ASSIGN, this is cardiovascular death (I00–I99), the first occurrence of hospitalization with coronary disease (I20–I25) or stroke (G45 and I60–I69), and Office of Population Censuses and Surveys: Classification of Interventions and Procedures Version 4 (OPCS-4) procedure codes (L29.5, L31.1, K40–46, K49, and K75 [procedures carotid endarterectomy, carotid angioplasty, coronary artery bypass graft, and percutaneous transluminal coronary angioplasty]). The SCORE2 outcome was defined more narrowly than ASSIGN as cardiovascular death (I10–I16, I20–I25, I46–I52, I60–I69, I70–I73, R96.0–R96.1 [excluding I51.4, I60, I62, I67.1, I68.2, and I67.5]), the first occurrence of nonfatal stroke (G45 and I60–I69), and the first occurrence of nonfatal myocardial infarction (I21–I22). For PCE, the outcome was defined as the first occurrence of nonfatal myocardial infarction (I21–I22), fatal coronary heart disease (I20–I25), or fatal or nonfatal stroke (G45 and I60–I69), again narrower than the ASSIGN outcome. More information on the derivation cohorts, outcomes, covariates, statistical approach, and model equations for each risk score are provided in Table S1.

### Statistical Analysis

Continuous variables are presented as median and interquartile range, and categorical variables are presented as absolute number (%). Ten-year estimated cardiovascular disease risks for each patient were calculated using the published risk models for ASSIGN, SCORE2, and PCE. The observed 10-year event rates were derived using Kaplan-Meier estimates to account for differing follow-up times among individuals.

Recalibration of the baseline survival was conducted to diminish over- or underestimation of risk. This was done by replacing the original 10-year baseline survival with the updated 10-year baseline survival derived from the GS:SFHS cohort. Recalibration was done in the whole cohort, not within each socioeconomic stratum. We evaluated the performance of the recalibrated and non-recalibrated risk scores by assessing measures of calibration and discrimination, stratified by socioeconomic deprivation status. Calibration refers to how closely the predicted 10-year risk agrees with the observed 10-year risk. Calibration plots were constructed using deciles of predicted risk scores. We evaluated the calibration intercept and slope of each plot. Furthermore, we calculated an observed versus predicted ratio by dividing the predicted risk by observed risk and evaluated whether the ratios differed between socioeconomic deprivation groups. We conducted a *Z* test to evaluate differences between predicted and observed risks, taking account of the uncertainty in the observed risk, and a *P* value <0.05 was considered statistically significant. Discrimination is the ability of the risk score to differentiate between patients who do and do not experience an event during the study period, and discrimination was assessed using the C-statistic. To further explore whether socioeconomic deprivation should be incorporated in cardiovascular risk scores, we fitted sex-specific Cox regression models to the GS:SFHS cohort using the same outcomes, model structure, and covariates as were used to derive the original ASSIGN, SCORE2, and PCE risk scores, but with socioeconomic deprivation status as an additional risk factor. The present article follows the Strengthening the Reporting of Observational Studies in Epidemiology (STROBE) guidelines.^26^ For the primary analysis, multiple imputation by chained equations was used to impute missing values of covariate data using fully conditional models including clinical characteristics and outcomes, and a single imputed dataset was selected. A complete-case sensitivity analysis was also performed. All statistical analyses were carried out in R (version 3.6.1). Key packages used were ‘*survival*’ to calculate Kaplan-Meier survival probabilities and fit relevant Cox proportional hazards models and ‘*ggplot2*’ to produce calibration plots. The R code is available on GitHub (https://github.com/leahpirondini/risk-score-calibration).

## Results

### Study Population

A total of 15 506 individuals (60.0% female, median 51 years of age) were included in our study. Individuals in the most deprived group were more likely to be female (group 1, 63.8% versus group 3, 57.9%), be current smokers (group 1, 30.1% versus group 3, 8.5%), and have diabetes (group 1, 4.0% versus group 3, 2.0%; Table 1). Despite being younger on average, individuals in the most deprived group had the highest incident risk of future cardiovascular events (Table 2). At 10 years, the cumulative incidence of cardiovascular death was 2.2%, 1.6%, and 1.4% for socioeconomic deprivation groups 1 (most deprived) to 3 (least deprived), respectively.

**Table 1.**
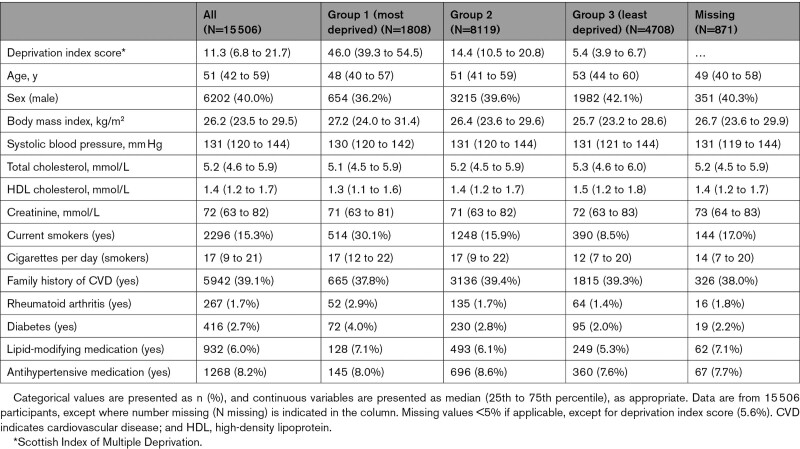
Clinical Characteristics of the Entire Study Population and Stratified by Socioeconomic Deprivation Status

**Table 2.**
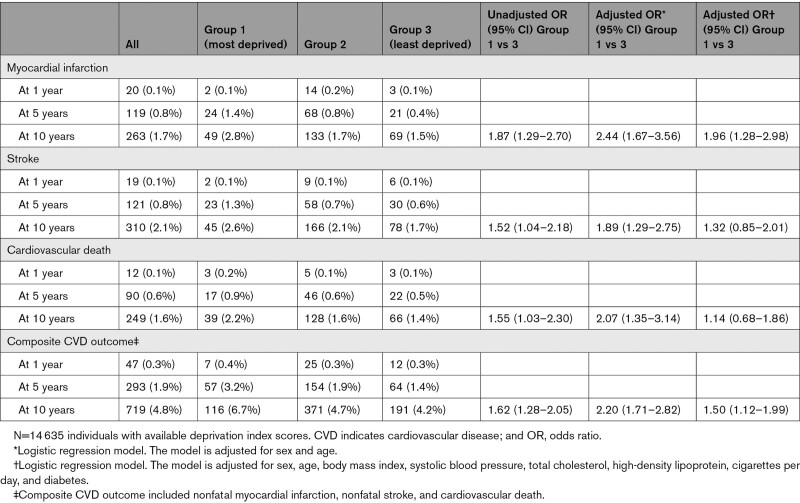
Incident Risk of Cardiovascular Events at 1, 5, and 10 Years

### Performance of Cardiovascular Risk Scores by Socioeconomic Deprivation Status

**Figure 1.**
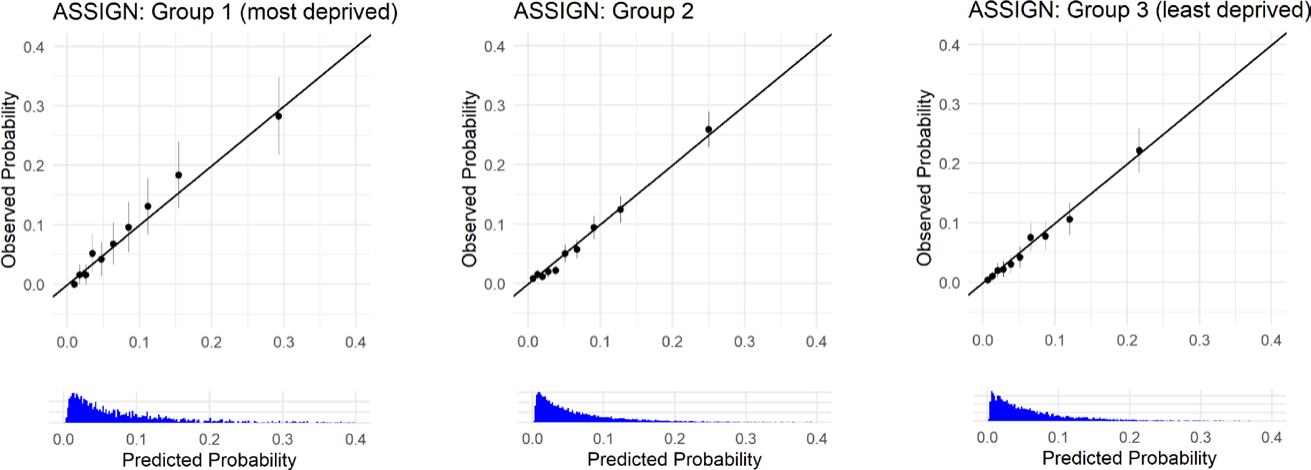
**Evaluation of the calibration of the recalibrated ASSIGN (assessing cardiovascular risk using SIGN [Scottish Intercollegiate Guidelines Network] guidelines to ASSIGN preventive treatment) risk score using the predicted and observed 10-year risk, stratified by socioeconomic deprivation status.** Each dot represents 1 decile of risk and is surrounded by the 95% CI.

**Figure 2.**
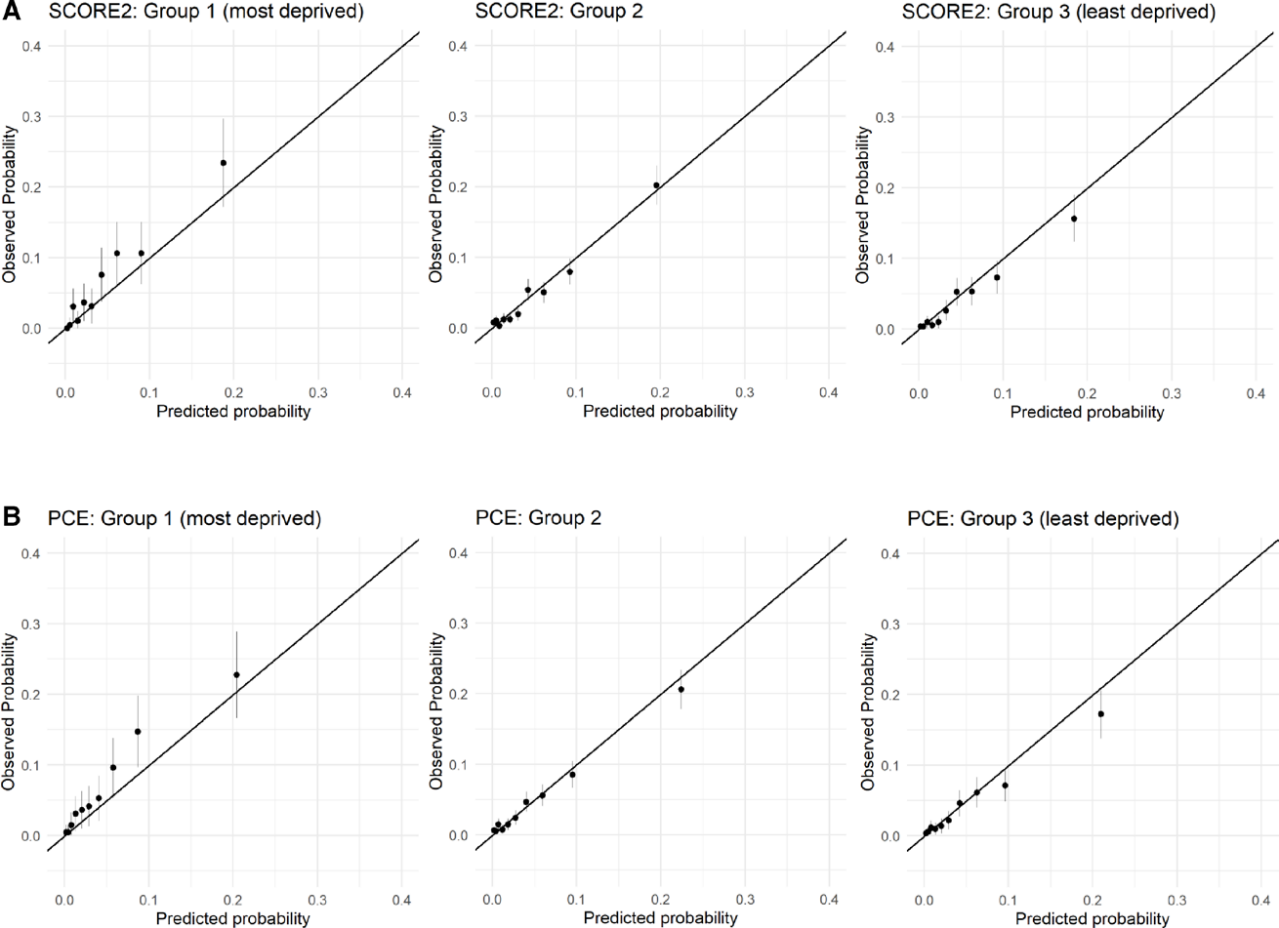
**Evaluation of the calibration of the recalibrated SCORE2 (Systematic Coronary Risk Evaluation 2 Algorithm) and Pooled Cohort Equations (PCE) risk scores using the predicted and observed 10-year risk, stratified by socioeconomic deprivation status. A**, SCORE2 and (**B**) PCE. Each dot represents 1 decile of risk and is surrounded by the 95% CI.

In the most deprived individuals, no statically significant difference was demonstrated between the observed versus predicted risk when using the recalibrated ASSIGN risk score (9.13% observed versus 8.39% predicted, *P*=0.256, Figure 1, Table 3). In contrast, risk scores based on the recalibrated SCORE2 and PCE models that did not include deprivation status significantly underpredicted risk in the most deprived (Figure 2, Table 3). For SCORE2, the observed risk was 6.43% in the most deprived individuals, whereas the predicted risk was 4.63% (*P*=0.001). Similarly, for PCE, the observed risk was higher at 6.69% than the predicted risk of 4.66% in those who were most deprived (*P*<0.001). A minimal and nonsignificant difference was demonstrated in the observed *versus* predicted risk when using the ASSIGN score in the least deprived (6.21% observed versus 6.45% predicted, *P*=0.478, Figure 1, Table 3). However, both SCORE2 and PCE risk scores significantly overpredicted the risk in the least deprived group (3.97% observed versus 4.72% predicted for SCORE2, *P*=0.007, and 4.22% observed versus 4.85% predicted for PCE, *P*=0.028, Figure 2, Table 3).

**Table 3.**
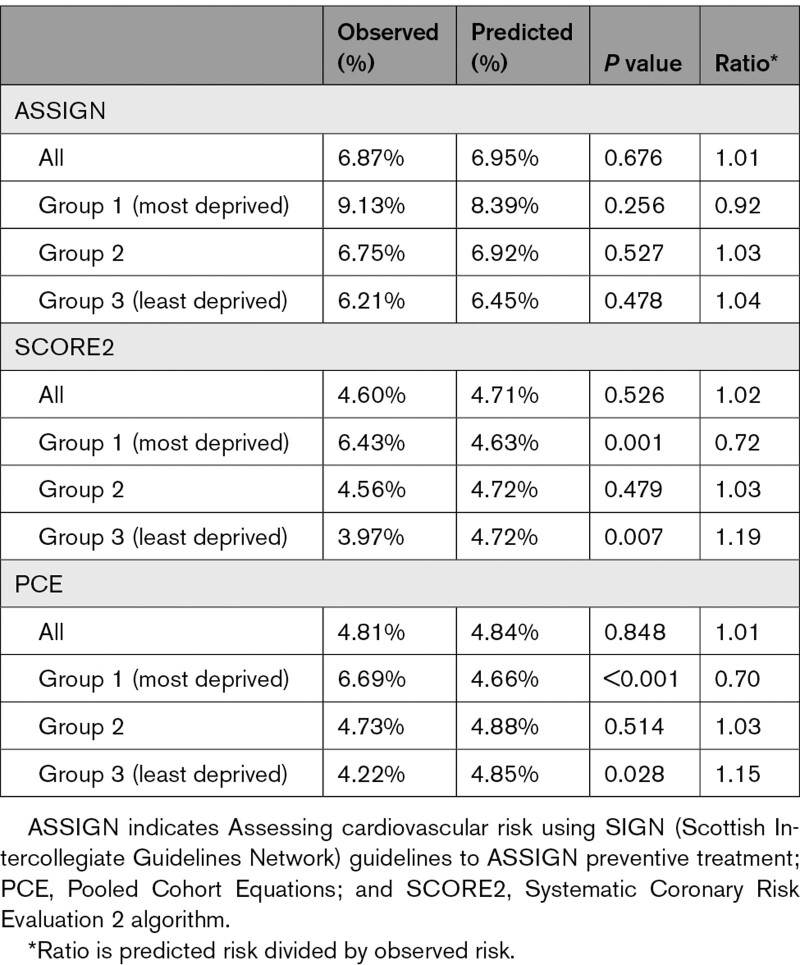
Observed and Predicted 10-Year Cardiovascular Risk Stratified by Socioeconomic Deprivation Status

In group 2, consisting of individuals in the middle deprivation categories, we demonstrated no statistically significant differences in observed versus predicted risk across all 3 risk scores (Table 3). For ASSIGN, the observed and predicted risks were 6.75% and 6.92% (*P*=0.527), respectively (Figure 1, Table 3). Both SCORE2 and PCE showed good agreement between observed and predicted risk (4.56% observed versus 4.72% predicted for SCORE2, *P*=0.479, and 4.73% observed versus 4.88% predicted for PCE, *P*=0.514, Figure 2, Table 3). The complete-case analysis yielded similar results (data not shown).

In addition, we studied the performance of the non-recalibrated cardiovascular risk scores across socioeconomic deprivation groups to evaluate the “raw” cardiovascular risk estimates of each risk score. None of the risk scores performed well before recalibration (Figure S1, Tables S2 and S3). All 3 risk scores showed good discrimination with slight differences between socioeconomic deprivation groups (Table S4). We further explored whether socioeconomic deprivation should be incorporated in cardiovascular risk scores by fitting sex-specific Cox regression models on GS:SFHS using the same framework (outcomes, covariates, and model structures) as the risk scores ASSIGN, SCORE2, and PCE model, and with socioeconomic deprivation status as an additional risk factor. All refitted risk models showed a significant contribution of the deprivation index score in the male-specific models (hazard ratio, 1.148 [95% CI, 1.092–1.206, ASSIGN], 1.176 [95% CI, 1.105–1.251, SCORE2], log hazard ratio 0.166 [95% CI, 0.111–0.221, PCE], Tables S5–S7).

## Discussion

We evaluated the effect of socioeconomic deprivation on the performance of 3 primary prevention cardiovascular risk scores that are widely used in clinical practice. The main finding of our study is that socioeconomic deprivation status is an important covariate in cardiovascular risk estimation systems. Risk scores (SCORE2 and PCE) that exclude socioeconomic deprivation in prediction modeling, meaningfully under- and overestimate the risk in the most and least deprived populations, respectively.^17,18^ As such, a substantial proportion of people living in more deprived communities at higher risk are likely to remain undertreated. The ASSIGN risk score—which includes socioeconomic deprivation in the risk prediction model—shows good performance in individuals living in the most and least deprived areas.^27^ Our analysis highlights the importance of incorporating a measure of socioeconomic deprivation status in risk estimation systems to reduce inequalities in health care provision for cardiovascular disease.

Low socioeconomic status is associated with worse cardiovascular outcomes,^1–5^ and our study showed that the magnitude of this association was strengthened after adjusting for sex and age. This is in line with published data showing that the differences in mortality rates between socioeconomic classes increased when age decreased, and observed mortality rates were highest in the youngest age groups.^28^ One in three premature deaths were attributable to socioeconomic inequalities and predominantly driven by cardiovascular disease.^29^ Our study shows that socioeconomic status is a largely unrecognized risk factor in primary prevention of cardiovascular disease. Incorporation of socioeconomic status into cardiovascular risk estimates is important to improving outcomes and closing the gap between the most and least deprived. The ASSIGN risk score was the first cardiovascular risk score developed that included socioeconomic deprivation status as a covariate to achieve equality for deprived individuals, and showed improvement—although marginal—in risk estimation compared with the Framingham Risk Score, which included only traditional risk factors.^10^ Similarly, QRISK2—which also includes socioeconomic deprivation as a covariate—showed higher accuracy compared with the Framingham Risk Score in the national QResearch database composed of 2.29 million patients.^30^ In addition, incorporation of socioeconomic status to the Framingham Risk Score was evaluated in the Atherosclerosis Risk in Communities study.^31^ When socioeconomic status was incorporated as an individual-based measure using income and education in the Framingham Risk Score, the bias towards deprived individuals disappeared.^31^ In line with these results, our study clearly demonstrates a bias to the most and least deprived areas towards under- and overestimating risk when using risk estimation systems that do not incorporate deprivation status. Furthermore, our analysis also shows that adding a deprivation index score to models using the same covariates as those of SCORE2 and PCE significantly contributed to the prediction of future cardiovascular events for men.

Cardiovascular risk estimation systems that do not incorporate deprivation status as a covariate may falsely classify the most deprived individuals at lower risk, potentially denying them the benefit of pharmacological and nonpharmacological primary prevention therapy. Similarly, the least deprived individuals may be falsely classified as high-risk, leading to potential overtreatment. A previous study modeling the potential effect of using risk estimation systems incorporating deprivation status showed that such a risk estimation system would result in initiation of lipid lowering therapy in 1 in 7 untreated individuals in the general population.^32^

None of the risk scores performed well when not recalibrated. Although the ASSIGN risk score has been tailored to the Scottish population, the estimated risk was 2-fold higher compared with the observed risk across the entire study population. The ASSIGN risk score was derived from a population in the 1980s in which the baseline risk was high.^10^ This baseline risk has fallen dramatically during the last 25 years,^5^ and the overestimation is most likely the result of an inaccurate baseline risk used in the risk equation.^5^ Our study shows that cardiovascular risk estimates could be optimized when risk scores are recalibrated using contemporary local data, which is in line with previous studies.^8,33–35^ Of the 3 risk scores evaluated, only SCORE2 acknowledged the need for recalibration and has used contemporary data to recalibrate their prediction models during development.^16,36^ However, we feel that the recalibration process should not be a static process. Cardiovascular risk estimation systems can be further optimized when a continuous recalibration system is in place. For example, the QRISK3 score is updated on an annual basis using contemporary local data to ensure that the baseline survival and mean of covariates used in risk equation reflect the target population that is being evaluated.^11^

Our study has several strengths. First, we used a contemporary cohort of >15 000 individuals that enabled us to evaluate the performance in a large group of deprived individuals and to recalibrate risk scores. Second, we had 10 years of follow-up available that allowed us to report an individual’s observed 10-year risk. Third, our cohort had adequate phenotyping at baseline for us to evaluate 3 cardiovascular risk scores that are commonly applied in clinical practice, and the outcomes for each risk score were matched to those used for derivation of the score as closely as possible.

We also acknowledge several limitations. First, although individuals were randomly invited to participate in GS:SFHS, the response rate was higher in less deprived individuals, and this might have tended to underestimate differences. Second, GS:SFHS predominantly includes individuals of a White background, and we cannot generalize our findings to individuals of other ethnic backgrounds. Third, we acknowledge that the social deprivation score used in our study is an area-based measure rather than the socioeconomic status of the individual introducing ecological bias, whereby an individual who lives in a more deprived area need not necessarily have to experience a high level of deprivation. This also highlights that deprivation on the basis of individual-level data may perform better than those based on geographical location. Furthermore, future work related to individual socioeconomic measures and risk prediction modeling is needed to unravel which component does particularly contribute to our observed findings. Fourth, our analysis showed that social deprivation was positively associated with cardiovascular outcomes for both men and women. However, the associations were stronger for men compared with women, and for women, the 95% CI crossed the line of unity. These observed differences in the effect estimate need further evaluation. Last, we limited our analysis to 3 cardiovascular risk scores. More risk scores have been developed over the years that are not included in our analysis, but we made the decision to focus particularly on those that are widely applied in practice across North America and Europe. We also did not include other risk estimation systems, including those with deprivation status, because of the lack of availability of model covariates or concordant outcomes.^11^

In conclusion, socioeconomic deprivation status is an important covariate in cardiovascular risk estimation systems. Risk scores that exclude socioeconomic deprivation under- and overestimate risk in the most and least deprived individuals, respectively. Our findings highlight the importance of incorporating socioeconomic deprivation status in risk estimation systems to reduce inequalities in health care provision for cardiovascular disease.

### Article Information

#### Acknowledgments

The authors are grateful to all the families who took part, the general practitioners and the Scottish School of Primary Care for their help in recruiting them, and the whole Generation Scotland team, which includes interviewers, computer and laboratory technicians, clerical workers, research scientists, volunteers, managers, receptionists, health care assistants, and nurses. D.M.K., L.P., J.G., D.P., S.J.P., and A.S.V.S. conceived the study and its design. D.M.K. and L.P. had access to the data and performed the analysis. D.M.K., L.P., and A.S.V.S. interpreted the data and drafted the article. All authors revised the article critically for important intellectual content and provided their final approval of the version to be published. All authors are accountable for the work.

#### Sources of Funding

Generation Scotland received core support from the Chief Scientist Office of the Scottish Government Health Directorates (CZD/16/6) and the Scottish Funding Council (HR03006). D.M.K. was supported by Health Data Research UK, which receives its funding from Health Data Research UK Ltd (HDR-5012) funded by the UK Medical Research Council, Engineering and Physical Sciences Research Council, Economic and Social Research Council, Department of Health and Social Care (England), Chief Scientist Office of the Scottish Government Health and Social Care Directorates, Health and Social Care Research and Development Division (Welsh Government), Public Health Agency (Northern Ireland), British Heart Foundation, and the Wellcome Trust. C.H. is supported by Medical Research Council University Unit Programme Grant MC_UU_00007/10 (Quantitative Trait Locus in Health and Disease). N.L.M. is supported by the British Heart Foundation through a Chair Award (CH/F/21/90010), a Programme Grant (RG/20/10/34966), and a Research Excellence Award (RE/18/5/34216). A.S.V.S. is supported by the British Heart Foundation through an Intermediate Clinical Research Fellowship (FS/19/17/34172). The funders had no role in the study and the decision to submit this work to be considered for publication.

#### Disclosures

Dr Shah’s institution has received honoraria from Abbott Diagnostics. Dr Sattar has received fees for consulting and speaking and honoraria from Amgen, AstraZeneca, Boehringer Ingelheim, Eli Lilly, Merck Sharp & Dohme, Novartis, Novo Nordisk, Pfizer, and Sanofi, and a research grant from Boehringer Ingelheim. Dr Mills has received personal fees from Abbott Diagnostics, Roche Diagnostics, Siemens Healthineers, and LumiraDx and has received grants awarded to the University of Edinburgh from Abbott Diagnostics and Siemens Healthineers outside the submitted work. The other authors report no conflicts.

#### Supplemental Material

Figure S1

Tables S1–S7

## Supplementary Material


