# Supplementary material for: Socioeconomic Deprivation: An Important, Largely Unrecognized Risk Factor in Primary Prevention of Cardiovascular Disease
Source: Circulation. 2022 Jun 24;146(3):240–8. doi: 10.1161/CIRCULATIONAHA.122.060042 (PMC9287096; doi:10.1161/CIRCULATIONAHA.122.060042)
Supplement: Supplementary file 1 [file cir-146-240-s001.pdf]

SUPPLEMENTAL MATERIAL

Socioeconomic deprivation: an important largely unrecognized risk factor in primary prevention of cardiovascular disease

Running title: Socioeconomic deprivation and CVD risk scores

Dorien M Kimenai, PhD[1*], Leah Pirondini, MSc[2*], John Gregson, PhD[2], David Prieto, PhD[3], Stuart J Pocock, PhD[2], Pablo Perel, PhD[3], Tilly Hamilton, BSc[1], Paul Welsh, PhD[4], Archie Campbell, MA[5], David J Porteous, PhD[5], Caroline Hayward, PhD[6], Naveed Sattar, MD, PhD[4], Nicholas L Mills, MD, PhD[1,7], Anoop SV Shah, MD, PhD[3]

[1] *BHF Centre for Cardiovascular Science, University of Edinburgh, UK*
[2] *Department of Medical Statistics, London School of Hygiene & Tropical Medicine, London, UK*
[3] *Department of Non-communicable Disease Epidemiology, London School of Hygiene & Tropical Medicine, London, UK*
[4] *Institute of Cardiovascular & Medical Sciences, University of Glasgow, Glasgow, UK*
[5] *Centre for Genomic and Experimental Medicine, Institute of Genetics and Cancer, University of Edinburgh, Edinburgh, UK*
[6] *MRC Human Genetics Unit, Institute of Genetics and Cancer, University of Edinburgh, Edinburgh, UK*
[7] *Usher Institute, University of Edinburgh, Edinburgh, UK*

[*]These authors contributed equally

Corresponding author:
Anoop SV Shah, MD, PhD
Department of Non-communicable Disease Epidemiology
London School of Hygiene and Tropical Medicine
London WC1E 7HT
United Kingdom

Email: anoop.shah@lshtm.ac.uk

Table S1. Description of ASSIGN, SCORE2 and PCE risk scores

ASSIGN	SCORE2	PCE
**Reference**		
Woodward et al. Adding social deprivation and family history to cardiovascular risk assessment: the ASSIGN score from the Scottish Heart Health Extended Cohort (SHHEC). *Heart* 2007;93:172–176. doi: 10.1136/hrt.2006.108167.	SCORE2 working group and ESC Cardiovascular risk collaboration. SCORE2 risk prediction algorithms: new models to estimate 10-year risk of cardiovascular disease in Europe. *European Heart Journal*. 2021;42(25):2439-2454. doi: 10.1093/eurheartj/ehab309.	Goff et al**.** 2013 ACC/AHA Guideline on the Assessment of Cardiovascular Risk A Report of the American College of Cardiology/American Heart Association Task Force on Practice Guidelines. *Circulation.* 2014;129:S49-73. doi: 10.1161/01.cir.0000437741.48606.98.
**Derivation and external validation cohorts**		
Derivation cohort: the Scottish Heart Health Extended Cohort (SHHEC). SHHEC includes overlapping studies. The Scottish Heart Health Study recruited random samples of men and women aged 40–59 years across 25 districts of Scotland from 1984 to 1987. The Scottish MONICA Project recruited in Edinburgh and north Glasgow in 1986, north Glasgow again in 1989 and 1995, ages 25–64 and 1992, ages 25–74.<br><br>External validation cohort: None.	Derivation cohort: individual-participant data from 44 cohorts included in the Emerging Risk Factor Collaboration (ERFC) and the UK Biobank (UKB). The ERFC has collated and harmonized individual-participant data from many long-term prospective cohort studies of CVD risk factors and outcomes. The UKB is a single large prospective cohort study with individual-participant data on approximately 500,000 participants aged >40 years recruited across 23 UK-based assessment centers during 2006–10, and followed-up for cause-specific morbidity and mortality through linkages to routinely available national datasets and disease-specific registers.	Derivation cohort: the Atherosclerosis Risk in Communities (ARIC) study, the Cardiovascular Health Study (CHS), and the Coronary Artery Risk Development in Young Adults (CARDIA) study, combined with applicable data from the Framingham Original and Offspring Study cohorts.<br><br>External validation cohort: None. Internal validation is applied using a 10x10 cross-validation technique.

	External validation cohorts: the MOnica Risk, Genetics, Archiving and Monograph (MORGAM) project, the Biomarker for Cardiovascular Risk Assessment in Europe (BiomarCaRE) consortium, the European Prospective Investigation into Cancer and Nutrition—cardiovascular disease (EPIC-CVD), CPRD, Heinz-Nixdorf Recall study (HNR), Estonian Biobank, HAPIEE study, HUNT study, DETECT study, and Gutenberg Health Study (GHS).	
	**Outcome**	
Deaths from cardiovascular causes (ICD-9 codes 390–459, ICD-10 codes I00-I99) or any hospital discharge diagnosis post-recruitment (potentially several per admission) for coronary heart disease (ICD-9 codes 410–414, ICD-10 codes I20-I25) or cerebrovascular disease (ICD-9 codes 430–438, ICD-10 codes G45, I60-I69), or for coronary artery interventions (CABG or PTCA).	Death from cardiovascular causes (ICD 9 codes 401-405, 410-414, 426-429, 430-438, 440-443, 798.1, 798.2, ICD-10 codes I10-16, I20-25, I46-52, I60-69, I70-73, R96.0-96.1), non-fatal stroke (ICD-9 codes 430-438, ICD-10 codes I60-69) and non-fatal myocardial infarction (ICD-9 codes 410, ICD-10 codes I21-I23).<br><br>The following ICD-9 and ICD-10 codes are excluded from death from cardiovascular causes: ICD-9 codes 426.7, 429, 430, 432.1, 437.3, 437.4, ICD-10 codes I51.4, I60, I62, I67.1, I68.2, I67.5).<br><br>The following ICD-9 and ICD-10 codes are excluded from death from non-fatal stroke	Death from coronary heart disease, fatal or non-fatal stroke and non-fatal myocardial infarction. Information on the ICD-9 and/or ICD-10 codes used to define the outcome is not provided in the original paper.

	endpoint: ICD-9 codes 429, 430, 432.1, 437.3, 437.4, ICD-10 codes, I60, I62, I67.1, I68.2, I67.5).	
**Covariates**		
• Age<br>• Total cholesterol<br>• HDL cholesterol<br>• SBP<br>• Diabetes<br>• Family history of CVD<br>• Cigarettes per day (for patients with Rheumatoid arthritis, 10 cigarettes per day are added)<br>• **Deprivation index score/10**	• Age<br>• Total cholesterol<br>• HDL cholesterol<br>• SBP<br>• Diabetes<br>• Smoking (Current or other)<br><br>• Total cholesterol * Age interaction<br>• HDL cholesterol * Age interaction<br>• SBP * Age interaction<br>• Smoking * Age interaction	• Age<br>• Age squared (females only)<br>• Total cholesterol<br>• HDL cholesterol<br>• SPB<br>• Diabetes<br>• Smoking (Current or other)<br><br>• Total cholesterol * Age interaction<br>• HDL cholesterol * Age interaction<br>• Smoking * Age interaction
**Statistical approach**		
Cox proportional hazards models fit within each sex.	Fine and Gray models stratified by cohort and fit within each sex.	Cox proportional hazards models fit within each sex and each ethnic group (white and African American).
**Male-specific risk equation**		

$h(t) = h_0(t)\exp(\beta_{age}Age + \beta_{tc}TC + \beta_{hdlc}HDLC + \beta_{sbp}SBP + \beta_{dm}DM + \beta_{family}Family + \beta_{cpd}CPD + \beta_{SIMD10}\frac{SIMD}{10})$	$h_k^s(t) = h_{0,k}^s(t)\exp(\beta_{age}Age + \beta_{tc}TC + \beta_{hdlc}HDLC + \beta_{sbp}SBP + \beta_{dm}DM + \beta_{smoke}Smoke + \beta_{age*TC}Age * TC + \beta_{age*HDLC}Age * HDLC + \beta_{age*SBP}Age * SBP + \beta_{age*DM}Age * DM + \beta_{age*smoke}Age * Smoke)$	$h(t) = h_0(t)\exp(\beta_{age}\log(Age) + \beta_{tc}\log(TC) + \beta_{hdlc}\log(HDLC) + \beta_{sbp}\log(SBP) + \beta_{dm}DM + \beta_{smoke}Smoke + \beta_{age*TC}\log(Age) * \log(TC) + \beta_{age*HDLC}\log(Age) * \log(HDLC) + \beta_{age*smoke}\log(Age) * Smoke)$
**Female-specific risk equation**		
$h(t) = h_0(t)\exp(\beta_{age}Age + \beta_{tc}TC + \beta_{hdlc}HDLC + \beta_{sbp}SBP + \beta_{dm}DM + \beta_{family}Family + \beta_{cpd}CPD + \beta_{SIMD10}\frac{SIMD}{10})$	$h_k^s(t) = h_{0,k}^s(t)\exp(\beta_{age}Age + \beta_{tc}TC + \beta_{hdlc}HDLC + \beta_{sbp}SBP + \beta_{dm}DM + \beta_{smoke}Smoke + \beta_{age*TC}Age * TC + \beta_{age*HDLC}Age * HDLC + \beta_{age*SBP}Age * SBP + \beta_{age*DM}Age * DM + \beta_{age*smoke}Age * Smoke)$	$h(t) = h_0(t)\exp\left(\beta_{age}\log(Age)\right) + \beta_{agesq}\log(Age)^2 + \beta_{tc}\log(TC) + \beta_{hdlc}\log(HDLC) + \beta_{sbp}\log(SBP) + \beta_{dm}DM + \beta_{smoke}Smoke + \beta_{age*TC}\log(Age) * \log(TC) + \beta_{age*HDLC}\log(Age) * \log(HDLC) + \beta_{age*smoke}\log(Age) * Smoke)$
**Additional information**		

• Variables centered at their mean values	• Variables centered at their mean values<br>• Rescaling factors used to scale individual predicted risks to the target population based on recent nationally representative estimates of incident cardiovascular disease and risk factor levels. Risk regions are divided into low risk, moderate risk, high risk and very high risk.	• Models presented above are for white males and white females. Ethnicity is not available in the GS:SFHS.

Table S2. Calibration intercept and slope of non-recalibrated and recalibrated cardiovascular risk scores

	All	Group 1 (most deprived)	Group 2	Group 3 (least deprived)
**Non-recalibrated risk score**				
ASSIGN				
Intercept	-1.1	-1.2	-1.2	-1.0
Slope	0.9	0.8	0.9	1.0
SCORE2				
Intercept	1.4	1.3	1.4	1.4
Slope	1.4	1.2	1.4	1.5
PCE				
Intercept	-0.6	-0.3	-0.6	-0.7
Slope	0.8	0.8	0.8	0.9
**Recalibrated risk score**				
ASSIGN				
Intercept	0.0	0.1	0.0	0.0
Slope	1.0	1.0	1.0	1.0
SCORE2				
Intercept	-0.1	0.1	-0.1	-0.2
Slope	1.0	0.9	1.0	1.0
PCE				
Intercept	-0.4	-0.1	-0.4	-0.5
Slope	0.9	0.8	0.9	0.9

Table S3. Observed and predicted 10-year cardiovascular risk of non-recalibrated risk scores stratified by socioeconomic deprivation status

	Observed (%)	Predicted before recalibration (%)	P-value	Ratio before recalibration*
**ASSIGN**				
All	6.87%	14.75%	<0.001	2.15
Group 1 (most deprived)	9.13%	18.05%	<0.001	1.98
Group 2	6.75%	14.68%	<0.001	2.17
Group 3 (least deprived)	6.21%	13.61%	<0.001	2.19
**SCORE2**				
All	4.60%	3.62%	<0.001	0.79
Group1 (most deprived)	6.43%	3.60%	<0.001	0.56
Group 2	4.56%	3.61%	<0.001	0.79
Group 3 (least deprived)	3.97%	3.63%	0.218	0.91
**PCE**				
All	4.81%	5.83%	<0.001	1.21
Group 1 (most deprived)	6.69%	5.64%	0.063	0.84
Group 2	4.73%	5.87%	<0.001	1.24
Group 3 (least deprived)	4.22%	5.84%	<0.001	1.38

*Ratio = predicted risk divided by observed risk.

Table S4. Discrimination of recalibrated cardiovascular risk scores

	All	Group 1 (most deprived)	Group 2	Group 3 (least deprived)
ASSIGN	0.779	0.760	0.785	0.770
SCORE2	0.796	0.773	0.801	0.802
PCE	0.793	0.781	0.798	0.794

Table S5. Estimated hazard ratios, 95% confidence intervals and p-values from a Cox proportional hazards model for composite CVD outcome, fitted to GS:SFHS cohort using the same structural form as that of the ASSIGN risk score

	**Male**		**Female**	
**Covariate**	**HR (95% CI)**	**P-value**	**HR (95% CI)**	**P-value**
Age	1.085 (1.077 to 1.093)	<0.001	1.095 (1.086 to 1.103)	0.001
Total cholesterol	1.140 (1.059 to 1.226)	<0.001	1.044 (0.965 to 1.129)	0.281
HDL	0.574 (0.458 to 0.720)	<0.001	0.602 (0.485 to 0.747)	0.001
SBP	1.006 (1.001 to 1.010)	0.011	1.009 (1.004 to 1.013)	0.001
Diabetes	1.826 (1.372 to 2.431)	0.001	1.856 (1.342 to 2.567)	0.001
Family history of CVD	1.209 (1.040 to 1.406)	0.013	1.049 (0.889 to 1.238)	0.572
Cigarettes per day	1.023 (1.015 to 1.031)	<0.001	1.036 (1.026 to 1.046)	0.001
**Deprivation index score/10***	**1.148 (1.092 to 1.206)**	**<0.001**	**1.052 (0.996 to 1.111)**	**0.069**
**Baseline 10-year survival**	0.937	#	0.974	#

# = not applicable. *Scottish Index Multiple Deprivation score. Abbreviations: CVD = cardiovascular disease, HDL = high-density lipoprotein, SBP = systolic blood pressure.

Table S6. Estimated hazard ratios, 95% confidence intervals and p-values from a Cox proportional hazards model for composite CVD outcome, fitted to GS:SFHS cohort using the same structural form as that of the SCORE2 risk score with social deprivation score included

	Male		Female	
**Covariate**	**HR (95% CI)**	**P-value**	**HR (95% CI)**	**P-value**
Age	1.427 (1.314 to 1.550)	<0.001	1.663 (1.517 to 1.824)	<0.001
Total cholesterol	1.233 (1.120 to 1.359)	<0.001	1.140 (1.024 to 1.268)	0.017
HDL	0.805 (0.694 to 0.933)	0.004	0.760 (0.655 to 0.883)	0.001
SBP	1.127 (1.005 to 1.264)	0.040	1.167 (1.028 to 1.325)	0.017
Diabetes	1.882 (1.253 to 2.827)	0.002	1.881 (1.118 to 3.164)	0.017
Smoking	1.472 (1.092 to 1.986)	0.011	2.230 (1.639 to 3.034)	<0.001
**Deprivation index score/10***	**1.176 (1.105 to 1.251)**	**<0.001**	**1.046 (0.971 to 1.127)**	**0.233**
Smoking*Age interaction	0.898 (0.798 to 1.011)	0.075	0.856 (0.748 to 0.979)	0.023
SBP* Age interaction	0.996 (0.947 to 1.047)	0.871	0.978 (0.933 to 1.026)	0.361
Total cholesterol* Age interaction	0.956 (0.920 to 0.994)	0.025	1.008 (0.966 to 1.052)	0.710
HDL* Age interaction	1.026 (0.958 to 1.099)	0.470	0.970 (0.910 to 1.034)	0.354
Diabetes* Age interaction	1.031 (0.838 to 1.268)	0.773	0.938 (0.770 to 1.144)	0.528
**Baseline 10-year survival**	0.946	#	0.971	#

# = not applicable. *Scottish Index Multiple Deprivation score. Abbreviations: HDL = high-density lipoprotein, SBP = systolic blood pressure.

Table S7. Estimated hazard ratios, 95% confidence intervals and p-values from a Cox proportional hazards model for composite CVD outcome, fitted to GS:SFHS cohort using the same structural form as that of the PCE risk score with social deprivation score included

	Male		Female	
**Covariate**	**Log HR (95% CI)**	**P-value**	**Log HR (95% CI)**	**P-value**
Log age	5.090 (4.450 to 5.730)	<0.001	5.510 (4.760 to 6.270)	<0.001
Log age squared	#	#	4.250 (2.490 to 6.010)	<0.001
Log total cholesterol	1.160 (0.685 to 1.630)	<0.001	0.460 (-0.141 to 1.060)	0.134
Log HDL	-0.831 (-1.200 to -0.467)	<0.001	-0.757 (-1.200 to -0.314)	<0.001
Log SBP	0.563 (-0.167 to 1.290)	0.131	0.814 (0.053 to 1.580)	0.036
Smoking	0.571 (0.341 to 0.800)	<0.001	0.840 (0.574 to 1.110)	<0.001
Diabetes	0.698 (0.373 to 1.020)	<0.001	0.487 (0.083 to 0.891)	0.018
**Deprivation index score/10***	**0.166 (0.111 to 0.221)**	**<0.001**	**0.055 (-0.009 to 0.120)**	**0.090**
Log total cholesterol* log age interaction	-4.100 (-6.230 to -1.980)	<0.001	-0.960 (-3.250 to 1.320)	0.408
Log HDL cholesterol* log age interaction	1.680 (-0.166 to 3.530)	0.074	0.478 (-1.350 to 2.310)	0.609
Current smoker* log age interaction	-1.630 (-2.780 to -0.475)	0.006	-0.849 (-2.130 to 0.432)	0.194
**Baseline 10-year survival**	0.958	#	0.980	#

# = not applicable. *Scottish Index Multiple Deprivation score. Abbreviations: HDL = high-density lipoprotein, SBP = systolic blood pressure.

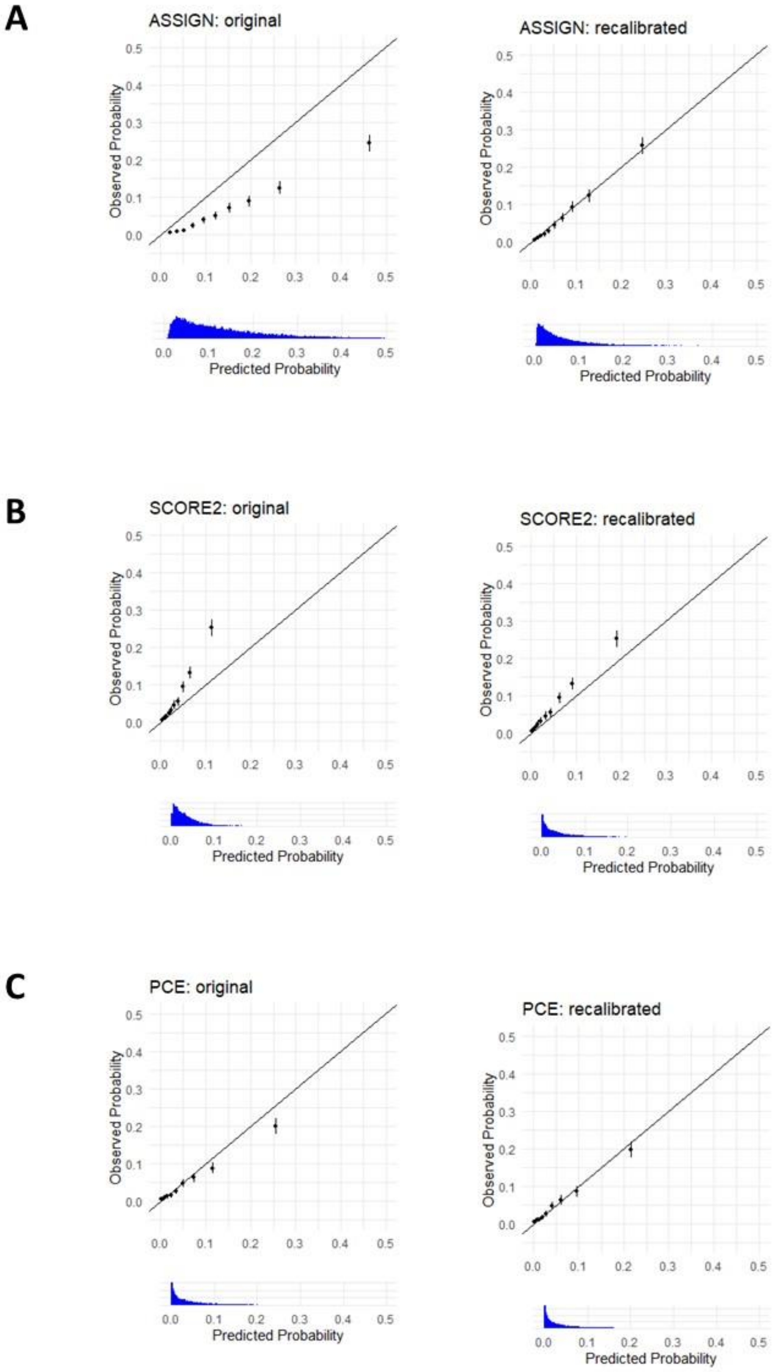

Figure S1. Evaluation of the calibration of the cardiovascular risk scores ASSIGN (Panel A), SCORE2 (Panel B) and PCE (Panel C) using the predicted and observed 10-year risk, before and after recalibration. Each dot represents one decile of risk and is surrounded by 95% confidence interval.
