# Supplementary material for: Socioeconomic Deprivation: An Important, Largely Unrecognized Risk Factor in Primary Prevention of Cardiovascular Disease
Source: Circulation. 2022 Jun 24;146(3):240–8. doi: 10.1161/CIRCULATIONAHA.122.060042 (PMC9287096; doi:10.1161/CIRCULATIONAHA.122.060042)
Supplement: Supplementary file 2 [file cir-146-240-s002.pdf]

Clinical perspective

What is new?

- We report the impact of socioeconomic deprivation on the performance of three primary prevention cardiovascular risk scores that are widely used in practice.
- Socioeconomic deprivation status is an important covariate in cardiovascular risk estimation systems, and risk scores that exclude socioeconomic deprivation under- and over-estimate risk in the most and least deprived individuals, respectively.

What are the clinical implications?

- Socioeconomic status is a largely unrecognized risk factor in primary prevention of cardiovascular disease.
- Our findings highlight the importance of socioeconomic deprivation status as a covariate that needs to be considered in addition to the traditional risk factors to promote equitable healthcare, particularly in those most deprived.
